# Navigating Grief: Understanding the Impact of Pregnancy Loss on Parental Attachment to the Second Child

**DOI:** 10.1192/j.eurpsy.2025.777

**Published:** 2025-08-26

**Authors:** C. M. Grego, A. Tarelho

**Affiliations:** 1 Psychiatry and Mental Health Department, Local Health Unit of Aveiro Region, Aveiro, Portugal

## Abstract

**Introduction:**

Pregnancy loss is a significant emotional experience that can shape subsequent parenting dynamics, particularly the attachment process to the next-born child. While attachment theory provides a framework for understanding how parents bond with their children, the effects of pregnancy loss on attachment to a subsequent child remain complex and understudied. This review aims to synthesize current research on the relationship between pregnancy loss and parental attachment to the next-born child, considering factors such as grief, coping mechanisms, and emotional healing.

**Objectives:**

The primary objective of this review is to examine existing literature to determine: (1) How pregnancy loss impacts parental attachment to a subsequent child; (2) The emotional, psychological, and contextual factors that influence the attachment process post-loss; (3) Gaps in the research and potential areas for future investigation.

**Methods:**

This review study systematically examines peer-reviewed articles, empirical studies, and theoretical papers published between 2000 and 2024 on the topic of pregnancy loss and subsequent child attachment. The databases used include PubMed and Google Scholar. Studies were included if they explored parental attachment post-loss, considered factors like grief and coping, and employed qualitative or quantitative measures of attachment. The literature was evaluated for methodological rigor and relevance to the study’s objectives.

**Results:**

The review identified consistent evidence that pregnancy loss can significantly affect attachment to the second child. Factors such as unresolved grief, heightened anxiety, and fear of loss contributed to difficulties in forming secure attachments. However, some parents demonstrated increased emotional investment in the second child as part of their healing process. The role of external support systems, such as counseling and social support, was identified as critical in mitigating attachment challenges. Additionally, research showed that fathers and mothers might experience attachment differently after loss, with cultural and individual factors influencing outcomes.

**Conclusions:**

This review underscores the complex and nuanced relationship between pregnancy loss and attachment to a subsequent child. While many parents experience heightened emotional challenges, supportive interventions can facilitate healthier attachment processes. Further research is needed to explore the long-term implications of these attachments and to develop targeted therapeutic strategies for parents navigating pregnancy after loss. The findings have significant implications for healthcare providers, offering guidance on how to support families during this critical period.

**Disclosure of Interest:**

None Declared

